# HaploPOP: a software that improves population assignment by combining markers into haplotypes

**DOI:** 10.1186/s12859-015-0661-6

**Published:** 2015-07-31

**Authors:** Nicolas Duforet-Frebourg, Lucie M. Gattepaille, Michael G.B Blum, Mattias Jakobsson

**Affiliations:** 1 Univ. Grenoble Alpes, TIMC-IMAG, Grenoble, F-38000 France; 2 CNRS, TIMC-IMAG, Grenoble, F-38000 France; 3 Department of Integrative Biology, University of California Berkeley, Berkeley, 94720-3140 California USA; 4 Department of Evolutionary Biology, Evolutionary Biology Centre, Uppsala University, Uppsala, Sweden; 5 Science for Life Laboratory, Uppsala University, Uppsala, Sweden

## Abstract

**Background:**

In ecology and forensics, some population assignment techniques use molecular markers to assign individuals to known groups. However, assigning individuals to known populations can be difficult if the level of genetic differentiation among populations is small. Most assignment studies handle independent markers, often by pruning markers in Linkage Disequilibrium (LD), ignoring the information contained in the correlation among markers due to LD.

**Results:**

To improve the accuracy of population assignment, we present an algorithm, implemented in the *HaploPOP* software, that combines markers into haplotypes, without requiring independence. The algorithm is based on the Gain of Informativeness for Assignment that provides a measure to decide if a pair of markers should be combined into haplotypes, or not, in order to improve assignment. Because complete exploration of all possible solutions for constructing haplotypes is computationally prohibitive, our approach uses a greedy algorithm based on windows of fixed sizes. We evaluate the performance of *HaploPOP* to assign individuals to populations using a split-validation approach. We investigate both simulated SNPs data and dense genotype data from individuals from Spain and Portugal.

**Conclusions:**

Our results show that constructing haplotypes with *HaploPOP* can substantially reduce assignment error. The *HaploPOP* software is freely available as a command-line software at www.ieg.uu.se/Jakobsson/software/HaploPOP/.

## Background

Molecular markers provide powerful approaches in forensic science and ecology to assign individuals into predefined populations [1, 2]. With the advent of new sequencing technologies, the number of available molecular markers in different species is rapidly increasing. At the same time, dense datasets tend to contain increasingly correlated markers because Single Nucleotide Polymorphisms (SNPs) that are physically close on a chromosome, often are in Linkage Disequilibrium (LD). Such correlations are usually perceived as a nuisance factor in statistical analyses since it violates a common assumption of independence among markers. This statistical nuisance can be overcome by pruning SNPs using for example the software *PLINK* [3]. However much information may be lost because of the pruning process. Another approach is to explicitly model the correlation between markers to control for LD [4–6], or to include the pruning process in the statistical analysis [7]. In addition, it has been shown that it can be useful to combine correlated markers into haplotypes to augment the information about population structure at a finer scale [8]. Such an approach is valuable for assignment methods when the level of genetic differentiation among groups is low [9].

Gattepaille and Jakobsson [10] introduced the Gain of Informativeness for Assignment (GIA), which is a statistic measuring the gain in information for population assignment by combining two markers into haplotypes. GIA is based upon an ancestry information criterion that measures to what extent a molecular marker is informative about population assignment [11]. GIA is defined as the difference between the ancestry information carried by two markers and the ancestry information carried by the haplotypes resulting from the combination of the two markers. Building haplotypes with GIA increases correct assignment to predefined populations [10]. However, a major combinatorial challenge arises when using GIA because of the prohibitively large number of pairs of markers that can be combined into haplotypes.

In this article, we present a new algorithm that efficiently uses GIA to build informative haplotypes for population assignment. The algorithm needs *reference* individuals whose population of origin is known. Based on these *reference* individuals, the algorithm uses GIA to construct informative haplotypes. To handle large numbers of markers, we provide a heuristic approach where only markers located within the same genomic region can be combined to form haplotypes. Combining markers into haplotypes is a recursive process so that haplotypes can result from the combination of two or more markers. The raw genotype data are recoded into multi-allelic haplotype data and the new data file containing both genotypic and haplotypic information can be used to assign individuals to populations based on for instance Principal Component Analysis, or model-based assignment approaches [12, 13].

Because the construction of haplotypes uses predefined populations, there is a risk of overfitting. For example if the evaluation of population assignment is performed with the same individuals that were used to construct the haplotypes, the assignment errors may be underestimated. Additionally, the constructed haplotypes can generate artificial population structure although there is no true stratification among the predefined populations. Both problems arise because the construction of haplotypes can exaggerate the differentiation among populations. To get a fair evaluation of population assignment, we implement a split-validation approach where we use different individuals to construct the haplotypes and to evaluate assignment [14]. Haplotypes are built using a subset of the individuals, consisting of a training set. The quality of population assignment can then be assessed using the remaining individuals (the validation set). If the individuals in the validation set cluster with individuals in the training set, there is evidence for some level of population structure, which may not have been detected based only on genotype markers.

Our new algorithm for combining markers into haplotypes is implemented in the software *HaploPOP*. The software is a command-line program written in C. We give examples of how to use *haploPOP* to perform population assignment with SNP data that were simulated from a population divergence model. We also show that *HaploPOP* improves assignment of individuals from Spain and Portugal using the POPRES dataset that contains 447,245 SNPs [15].

## Methods

### Gain of informativeness for assignment

The *Gain of Informativeness for Assignment* (*GIA*) is a one-dimensional statistic that provides a criterion to decide whether markers should be combined into haplotypes in order to improve population assignment [10]. It is based on the *Informativeness for Assignment* (*IA*) statistic, which measures how informative a marker is for assigning individuals to different populations [11]. The more different the allele frequencies are in a set of predefined populations, the more informative the marker is to assign individuals of unknown origin to their source population, and the larger is the *IA* statistic. Denoting by *K* the number of populations, by *N* the number of alleles of the marker under consideration, by $p_{j}^{(i)}$ the frequency of allele *j* in population *i*, and by $\overline {p}_{j}$ the average frequency of allele *j* across all populations, the *IA* statistic is computed as follows [11]

(1) $$ \textit{IA} = \sum\limits_{j=1}^{N}\left(-\overline{p}_{j}\log \overline{p}_{j} + \sum\limits_{i=1}^{K}\frac{p_{j}^{(i)}}{K}\log p_{j}^{(i)}\right).  $$

Given two multi-allelic markers *M*_1_ and *M*_2_, the question is whether combining *M*_1_ with *M*_2_ into a haplotype marker *H* improves the assignment of individuals to predefined populations. *GIA* computes the difference between the informativeness for assignment of *H* and the sum of the informativeness of *M*_1_ and *M*_2_

(2) $$ \textit{GIA} = \textit{IA}(H)-(\textit{IA}(M_{1})+\textit{IA}(M_{2})).  $$

If *GIA* is positive, it suggests that population assignment is improved by considering haplotype *H* instead of using the two markers separately. However, if *GIA* is negative, there is no advantage of combining the two markers into a haplotype. In particular, it can be shown that if the two markers are in linkage equilibrium, *GIA* is expected to be non-positive [10].

### Maximizing the informativeness for assignment

We assume that genotype data are available for *n* individuals at *l* molecular markers (*M*_1_,...*M*_*l*_). We also assume that the dataset has been phased, where all individuals have been phased together in one go to avoid introducing any haplotype difference due to phasing (note that there may still be switch errors from the phasing, but these should affect all individuals similarly). The approach implemented in the software *HaploPOP* builds a set of haplotypes that increases the total informativeness for assignment contained in the genotype data. To find the optimal haplotype set *Γ*_0_, we address the maximization problem

(3) $$  \left\{ \begin{array}{l} \displaystyle \Gamma_{0} = arg \max_{\Gamma} \sum\limits_{H \in \Gamma} \textit{IA}(H) \\ \Gamma \in Part(M_{1} \dots M_{l}) \end{array} \right.  $$

where *P* *a* *r* *t*(*M*_1_...*M*_*l*_) is the set of all possible partitions of the *l* markers. The number of partitions in a dataset of *l* markers is given by Bell’s number [16]. Because this number is large, we cannot evaluate the objective function for all possible partitions. A commonly used heuristic is to apply a greedy strategy, although it can perform arbitrarily good. In the case of increasing Informativeness for Assignment, the resulting haplotypes always provide genetic data with augmented informativeness. Because the cost of this algorithm increases rapidly with the number of genetic markers, we limit potential combinations of markers within windows of fixed size.

### Algorithm

In a first step, the algorithm constructs haplotypes from the phased genotype file of individuals with known origin and returns a haplotype coding file that provides the correspondence between haplotypes and initial markers. This is the LEARN option of *HaploPOP*. The construction of haplotypes is constrained by a predefined window-size. The set (*M*_1_,…*M*_*l*_) of markers is divided into subsets of contiguous markers corresponding to the genomic windows. Haplotypes are constrained to be combinations of markers of the same window. The window size is chosen by the user and can be defined based on number of markers, on base pairs, or genetic distance. By choosing genetic distances, one can account for non-uniform recombination rates. In every window, the *GIA* statistic is computed for all pairs of markers, and the pair with the greatest *GIA* value is merged to form a haplotype. Combinations proceed recursively until there is no pair of markers for which *GIA* >0 (or a certain positive user-defined threshold). A particular haplotype-loci formed by a combination of markers is thereafter treated as a (potentially multi-allelic) marker of the particular window and can be combined with other markers in a recursive manner.

We denote by *n* the number of *reference* individuals whose population of origin is known, and by *l* the total number of initial markers. The greedy algorithm proceeds as follows:

1. divide the 2*n*×*l* data matrix in contiguous windows.
2. for every window do
  - Calculate *GIA* for all pair of markers.
  - while for all markers *M* and *M*’, $\displaystyle {max}_{M, M^{\prime }} (\textit {GIA}(M, M^{\prime }) > 0)$, do
    i. $\displaystyle (M_{0},M^{\prime }_{0}) = {argmax}_{M, M^{\prime }} \textit {GIA}(M,M^{\prime }) > 0$.
    ii. Combine the markers *M*_0_ and $M^{\prime }_{0}$ to form a haplotype marker *H*_0_.
    iii. Remove the *GIA* statistics involving *M*_0_ and $M^{\prime }_{0}$ and compute the new *GIA* statistics with pairs of markers that include *H*_0_.

At every end of the inner loop, the algorithm partitions the markers into a set of haplotypes that increases the score of the objective function (3). It stops when no additional pairwise combination improves the total score of the partition. A warning is raised when the number of haplotype-alleles reaches the number of chromosomes 2*n* making haplotypes useless because they become private to every individual and do not provide any useful information for assignment.

In a second step, *HaploPOP* combines the SNPs in the initial genotype file into haplotypes according to the combinations of markers constructed at the first step, and generates the haplotype data file. The genotype file can contain individuals of unknown origin that the user is trying to determine, as well as the individuals of known or suggested origin used to construct the haplotypes. This corresponds to the APPLY function of *HaploPOP*. When two markers are combined, the resulting haplotype-alleles are coded in a range from 0 to the number of haplotype-alleles minus one, in order of appearance in the list of individuals.

### Window size

A key parameter of the method is the window size. This parameter is important for both speed of the algorithm and level of informativeness of the haplotypes. The choice of window size governs the number of operation performed by the algorithm. In the case of a fixed window of *S* markers, the number of windows is *n*_window_=*l*/*S*, and the cost *C* of the algorithm in number of operations is

(4) $$ C(n_{window}, S, n, K) = O(n_{window}\times(2nS^{2}K + S^{3}))  $$

where *K* is the number of populations in the data. The algorithm scales very well for genome wide datasets, since for a given window size *S*, the cost of the algorithm is proportional to the number of markers *l* in the data. The term proportional to *S* ^3^ corresponds to the iterative maximum search in all possible pairs of the window. The term *S* ^3^ is an upper bound for a search that is done in the worst case *S* times in a matrix of size *S*×*S* or less.

In the event of choosing a large window size, there may be a large number of haplotype-alleles, which could fit closely to the distribution of haplotype-alleles of individuals in the training set. Such a set of haplotypes would likely perform poorly for other sets of individuals from the same reference population, and reduces accuracy of population assignment. We refer to this phenomenon as overfitting. Limiting the size of the window is one way to avoid overfitting. We demonstrate in the Results section that the window size has a strong impact on the performance of the created combinations of markers, and an optimal value generally exists. The optimal window size depends on multiple factors, including the effective population sizes of the investigated groups and the extent of Linkage Disequilibrium in the groups.

### Split-validation

To validate the gain in assignment accuracy provided by the constructed haplotypes, we implement a split-validation technique [14]. For each population, we randomly split the set of individuals into two subsets consisting of the training subset used to learn the haplotypes and the validation subset used to compute assignment accuracy. It is important that the division between validation and training set is done after phasing. Phasing performed on the two datasets separately could introduce haplotypic differences and weaken the informativeness for assignment of the haplotypes built by the algorithm. To assign individuals to populations, we use Principal Component Analysis (PCA) as implemented in the software EIGENSOFT [12]. For each of the constructed haplotype-loci, we enumerate all haplotype-alleles present in the dataset. We use a presence/absence coding for each haplotype-allele. In particular, we add one column per haplotype-allele and note 1 for a chromosome carrying the allele, and 0 otherwise. The number of principal components we consider equals the number of populations used for constructing the haplotypes minus one [12]. We determine the PC axes using individuals from the training and the validation set. For each individual of the validation set, we compute Euclidean distances on the PC space between this individual and the barycentric coordinates of each population computed from the training set of individuals. We assign individuals to the population that has the closest barycenter. Because the origins of individuals in the validation sets were known for all examples (see below), we can measure the number of incorrectly assigned individuals in these examples. Note that the assessment of individuals to populations depends on the assignment procedure itself (here we use PCA) and that different assignment procedures may lead to different assignment errors (see [10] for a comparison of different assignment strategies). However, since we are primarily interested in the comparison between assignment using the raw genotype data and assignment using combined markers found with *HaploPOP*, we focus on a single assignment approach based on PCA. With this assignment approach each haplotype-allele is treated as a unique allele with the same relationship to all other alleles.

## Results and discussion

We evaluate the performance of the approach and the *HaploPOP* software on both simulated and empirical data.

### Application to simulated data

We evaluated the assignment approach and the *HaploPOP* software for simulated data generated by the software *ms* [17]. We simulated 200 kb sequences from a 3-population divergence model. We set the effective population sizes of all populations to *N*_*e*_=1,000, the mutation rate to *μ*=0.012, so that *θ*=48, and we considered a sample of 100 individuals in each population. The population divergence between population 2 and population 3 was set to occur at *T*_1_=0.025 coalescent time units (or 100 generations) in the past and the population divergence between population 2 and population 1, was set to occur at *T*_2_=0.05 coalescent time units (or 200 generations) in the past. We generated four datasets for a hypothetical 200 kb region with effective recombination rates of *ρ*=30,60,120, and 240, and replicated this procedure 10 times for each value of the recombination rate.

**Fig. 1:**
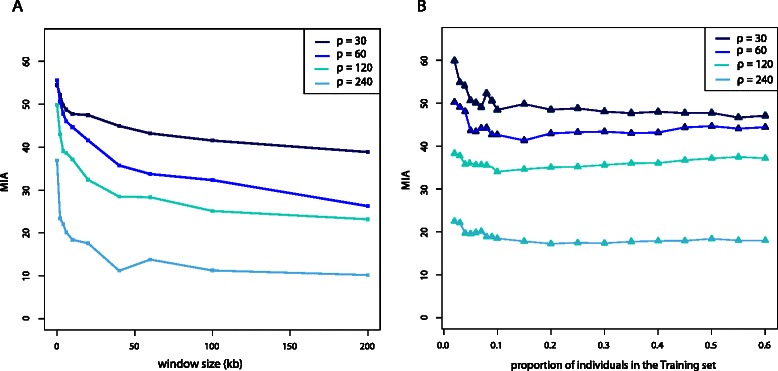
Mean percentage of Incorrect Assignment (MIA) for simulated data from a divergence model with 3 populations (see main text for details on simulations). Panel **a**: *x* axis represents the window sizes. Note that window size = 1 corresponds to using SNP genotype data to assign individuals to populations. Panel **b**: *x* axis represents the proportion of individuals in the samples that are used in the training set. The mean incorrect assignment of individuals is evaluated with individuals from the validation set that were not used to construct the haplotypes

To assess the assignment accuracy provided by the haplotypes constructed by *HaploPOP*, we used a split-validation technique. The training set and the validation set contained each 50 randomly chosen individuals in each population. Figure 1 a shows that assignment accuracy improves by constructing haplotypes. The assignment error decreases as the window size increases (up to a certain level). However, most of the improvement occurs when moving from genotypes to haplotypes spanning up to 50 kb (Fig. 1 a). Compared to the error of assignment obtained with genotype data, constructing haplotypes decreases the error by 20−70 *%* depending on the recombination rate. Constructing haplotypes for 200 kb windows compared to 50 kb windows only reduces the error by at most an additional 8 *%*.

Furthermore, we find that the mean incorrect assignment is lower with greater recombination rates. This emphasizes the fact that strongly correlated polymorphisms tend to carry less information for assignment than the same number of independant polymorphisms. Since simulated sequences have on average the same number of SNPs, sequences with a greater recombination rate carry more informativeness for assignment.

For a fixed window size of 50 kb, we construct the haplotypes for different sizes of training sets ranging from 2 to 60 individuals. When comparing the mean incorrect assignment of individuals from the validation set, we find a decay of MIA with increasing numbers of individuals in the training set for all recombination rates. However, when using a fraction of individuals greater than 10 *%* of the overall population, the change in MIA is minimal (Fig. 1 b). Hence, even a fairly small fraction of individuals in a sample can be used to accurately train the algorithm.

### Application on human data

**Fig. 2:**
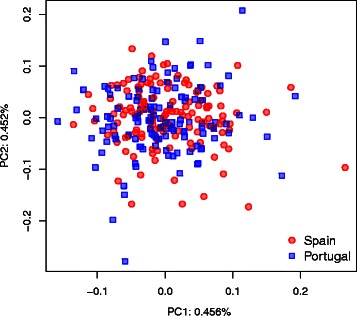
Principal Component Analysis on 447,245 SNPs for Spanish and Portuguese samples from POPRES

**Fig. 3:**
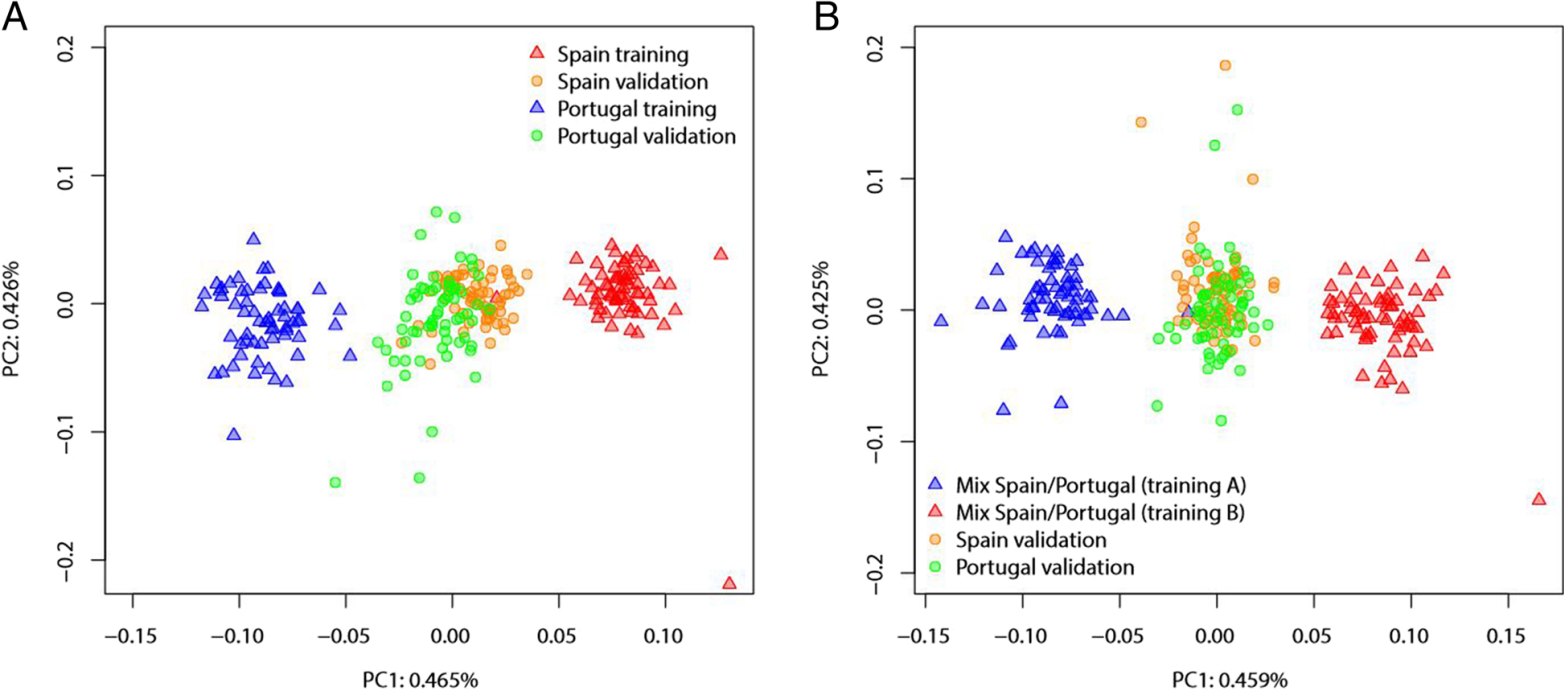
Principal Component Analysis of the Spanish and Portuguese samples from POPRES using the haplotypes found with *HaploPOP*. The haplotypes were built from 447,245 SNPs using a window size of 150 kb. For constructing haplotypes, the training sets consist of the Portuguese and Spanish individuals (Panel **a**) or a mix of Portuguese and Spanish individuals in both sets ‘A’ and ‘B’ (Panel **b**)

We investigate to what extent constructing haplotypes with *HaploPOP* improves population assignment of respectively 133 and 125 self-reported Spanish and Portuguese individuals from the POPRES dataset, which contains 447,245 SNPs [15]. We first phased the data using *fastPhase* [10, 18]. No pruning of SNPs were performed because our aim is to capture the information for assignment for all markers, including markers in LD. All markers were therefore retained and used to build haplotypes. Considering the first two PCs based on all SNP-genotype data, we found that the two populations cannot be distinguished (Fig. 2). A thorough PCA exploration of all European individuals of the POPRES collection was further unable to distinguish between Spanish and Portuguese individuals [19]. Using *HaploPOP*, we constructed the haplotypes that are informative to discriminate between Spanish and Portuguese individuals. We randomly selected 67 Spanish individuals and 63 Portuguese individuals for constructing the training set. We then performed PCA based on the haplotype markers generated by *HaploPOP* and compute the PC scores for the individuals from the training set and the validation set (Fig. 3 a). On PC1, the Spanish and Portuguese samples from the training set are clearly separated. By contrast, the 66 Spanish individuals and 62 Portuguese individuals from the validation set overlap but a large majority of these individuals are pulled in the direction of their population of origin.

To show that the population labels, Portugal and Spain, correspond to true population differentiation, we generated a control training set. We arbitrarily assign each individual of the training set to a label A or B and by construction, individuals labeled by A (or B) contain both individuals from Spain and Portugal. Using this training set of half the individuals in the dataset, we learn the haplotypes that are informative for discriminating between A and B. Using the validation set, we find that the haplotypes learned with populations A and B cannot distinguish between Spanish and Portuguese ancestry (Fig. 3 b). This analysis shows that the above demonstrated separation between Spanish and Portuguese individuals corresponds to true population differentiation and that the separation is not a consequence of overfitting.

These two examples emphasize two important features of *HaploPOP*. First, the haplotypes constructed based on the training set are very efficient in separating individuals of the training set, regardless of any true stratification between candidate populations. Comparing only the individuals from the training set on a PC plot can either lead to the wrong conclusion that two populations can be distinguished (Fig. 3 b) or at least exaggerate the ability to distinguish between the two populations (Fig. 3 a) because of overfitting. Second, evidence for population structure comes from the ability of the constructed haplotypes to distinguish between individuals that were not used in the training process. If a validation set of individuals can be assigned to the candidate populations, it is a good indication of fine-scale level of stratification between the candidate populations that might be difficult to detect using SNPs only.

**Fig. 4:**
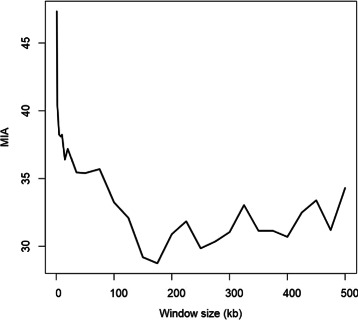
Mean percentage of Incorrect Assignment (MIA) when distinguishing the Spanish and Portuguese samples from POPRES. The error is evaluated with a split-validation approach

We show that two populations that were not distinguishable with raw genotype data can be separated based on haplotypes. This highlights how *HaploPOP* can be used to study samples where prior belief suggests that there is population structure but SNP-genotype data fail to detect it. When computing the error for assignment of the validation set for the POPRES data, we find that there is an optimal window size (Fig. 4) at which the assignment error can be reduced by 45 %. Intuitively, combining SNPs into haplotypes can only improve the power to assign individuals to groups up to some level: for too large window sizes, we run into overfitting problems where trained haplotypes are well suited to separate the particular individuals of the training set but not of the individuals in the validation set. The optimal value of the window size depends on many factors, such as the extent of linkage disequilibrium in the groups, or the degree of genetic differentiation between groups. The strategy we advocate for choosing the window size is to try different window sizes and to find the minimal assignment error as estimated with a split-validation approach. Such a strategy is computationally costly and requires for each of the chosen window size a run of *HaploPOP*, where the cost will be dependent of the window size *S* as described in equation (4).

Recently, many model-based methods have been proposed to assign unlabeled individuals to populations [13, 20–22]. These methods can be used together with *HaploPOP* to reduce the proportion of incorrect assignment, as it is shown with the software *Structure* [23] in a previous article [10]. In this article we focus on using Principal Component Analysis and, from a statistical point of view, model-based approaches and PCA are related [20]. In the case of assigning individuals to labeled populations, we expect that most of these methods will result in similar assignment accuracy.

## Conclusions

In this article, we present a new algorithm that uses the *GIA* statistic to construct haplotypes with a window-based approach. The algorithm is implemented in the command-line software *HaploPOP*. The software allows users to apply a 2-step procedure. First, *HaploPOP* constructs haplotypes that are informative about population assignment from a training set of individuals. Second, *HaploPOP* recodes the genotype data to haplotypes. These new haplotype data can then be used to assign unknown individuals to candidate populations or investigate fine-scale population structure using e.g. PCA. We have shown how constructing haplotypes with *HaploPOP* can substantially reduce mis-assignment of individuals to candidate populations. For SNP data simulated in a 3-population divergence model, the assignment-error was reduced by 20 *%* to 70 *%*. Using the 447,245 SNPs of the POPRES data, the assignment-error was reduced by 45 *%* when trying to distinguish Portuguese from Spanish individuals.

Constructing Haplotypes with *HaploPOP* is a promising approach to assign individuals into populations in forensic science and ecology. It can also confirm prior belief about fine-scale population structure which is a main confounding factor for association studies with rare variants [24].

## Availability and requirements

Linux and windows versions of the software are available at: www.ieg.uu.se/Jakobsson/software/HaploPOP/.
